# Transition from continental rifting to oceanic spreading in the northern Red Sea area

**DOI:** 10.1038/s41598-021-84952-w

**Published:** 2021-03-10

**Authors:** Sami El Khrepy, Ivan Koulakov, Taras Gerya, Nassir Al-Arifi, Mamdouh S. Alajmi, Ayman N. Qadrouh

**Affiliations:** 1 Natural hazards and mineral resources chair, Geology and Geophysics Department, King Saud University, P.O. Box 2455, Riyadh, 11451 Saudi Arabia; 2 Seismology Department, National Research Institute of Astronomy and Geophysics (NRIAG), Helwan, 11421 Egypt; 3 Trofimuk Institute of Petroleum Geology and Geophysics SB RAS, Prospekt Koptyuga, 3, Novosibirsk, Russia 630090; 4 Novosibirsk State University, Pirogova 2, Novosibirsk, Russia 630090; 5 Institute of Volcanology and Seismology FEB RAS, Piip Boulevard, 9, Petropavlovsk-Kamchatsky, Russia 693006; 6 Department of Earth Sciences, ETH Zurich, Sonneggstrasse 5, 8092 Zurich, Switzerland; 7 King Abdulaziz City of Science and Technology, Riyadh, Saudi Arabia

**Keywords:** Geodynamics, Geophysics, Seismology

## Abstract

Lithosphere extension, which plays an essential role in plate tectonics, occurs both in continents (as rift systems) and oceans (spreading along mid-oceanic ridges). The northern Red Sea area is a unique natural geodynamic laboratory, where the ongoing transition from continental rifting to oceanic spreading can be observed. Here, we analyze travel time data from a merged catalogue provided by the Egyptian and Saudi Arabian seismic networks to build a three-dimensional model of seismic velocities in the crust and uppermost mantle beneath the northern Red Sea and surroundings. The derived structures clearly reveal a high-velocity anomaly coinciding with the Red Sea basin and a narrow low-velocity anomaly centered along the rift axis. We interpret these structures as a transition of lithospheric extension from continental rifting to oceanic spreading. The transitional lithosphere is manifested by a dominantly positive seismic anomaly indicating the presence of a 50–70-km-thick and 200–300-km-wide cold lithosphere. Along the forming oceanic ridge axis, an elongated low-velocity anomaly marks a narrow localized nascent spreading zone that disrupts the transitional lithosphere. Along the eastern margins of the Red Sea, several low-velocity anomalies may represent crustal zone of massive Cenozoic basaltic magmatism.

## Introduction

The transition from continental rifting to oceanic spreading is a crucial yet partly enigmatic stage that determines the birth and further evolution of an oceanic basin. This transition has been broadly investigated using two dimensional (2D) and three-dimensional (3D) numerical geodynamic models. Both abrupt and gradual lithospheric extension has been suggested depending on the initial thermal-rheological structure of the continental lithosphere^1–5^. However, direct geophysical observations of the evolution of deep lithospheric structures are rare because of the limited availability of appropriate transitional settings and logistical problems. Among the most spectacular sites on Earth where such a transition can be directly observed is the Red Sea basin, particularly, its northern part where different stages of the process are simultaneously active. More than 50 years of multidisciplinary research^6–8^ (see also the overview in the supplement) has produced a comprehensive geological-geophysical database of the Red Sea region that enables testing of geodynamic hypotheses. However, previous geophysical studies did not provide sufficient spatial resolution for the deep lithospheric structures beneath the Red Sea to corroborate or disprove the existing concepts and test predictions from numerical geodynamical models.

The Red Sea is part of the large “Afro-Arabian rift system” that propagates from the Dead Sea to Mozambique^9^ and is considered among the youngest oceanic spreading zones in the world. It has formed as a result of the northeast displacement of the Arabian Plate with respect to the African Plate^10^ with the relative divergence varying from 10 to 16 mm/year^11,12^. The opening of the Red Sea began approximately 30 Ma because of the rupture of the Arabian–Nubian Shield, which may have been triggered by flood basalt volcanism caused by the Afar plume impingement on the non-uniformly stressed continental lithosphere^4,7,13^.

**Figure 1:**
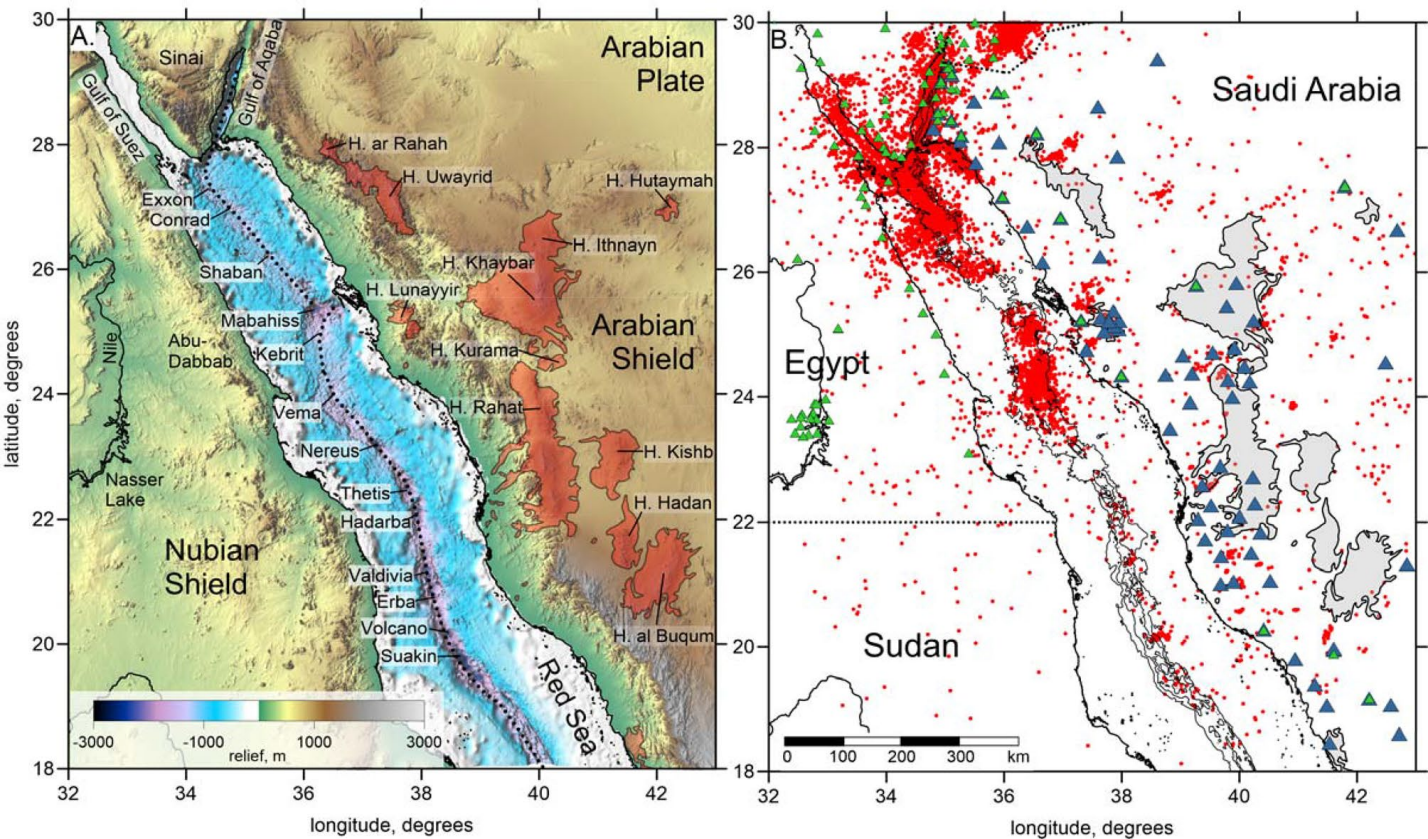
(**A**) Topography in the study area (http://www.marine-geo.org). Red areas highlight the Harrats (Cenozoic basaltic fields); the dotted line indicates the axis of the Red Sea spreading/rifting. The names of the major deeps (depression points) in the Red Sea are shown. (**B**) Distribution of data used in this study: red points are events, blue triangles are Saudi Arabian stations, and green triangles are Egyptian stations and stations reporting to the ISC that were used in the previous tomographic studies^21,22^. Dotted lines indicate political boundaries. The images have been produced using the Surfer Golden Software 13 (https://www.goldensoftware.com/products/surfer).

The opening of the southern part of the Red Sea (to the south of 20°N) presently occurs in the form of oceanic spreading^7^. Clearly identified linear magnetic anomalies around a distinct axial trough indicate that oceanic crust has developed here for at least 5 Ma^8,14–16^. However, in the northern half of the Red Sea, the lithospheric extension time is shorter and the extensional mechanism is not completely understood. The axial trough is not as clear here as it is in the southern part^6^ and it forms a series of discrete depressions termed “deeps,” some of which are shown in Fig. 1A. No clear linear magnetic anomalies can be identified in the northern Red Sea to the north of 22°N ^6,8,16^, which can be interpreted as evidence of continental extension. On the other hand, the crust in this part of the basin appears to be composed of a complex alternation of gabbroic and basaltic dikes, some of which have deep mantle origin and no continental crust contamination^17^. Furthermore, the estimated volume of the crustal material forming the Red Sea depression appears to be larger than the value following from the reconstruction based on correlating geological structures on the African and Arabian sides^18^. This means that some new crust needs to appear in some manner to satisfy this balance. Thus, both mechanisms of continental crust extension (i.e., stretching and faulting) and magmatic additions may occur in the northern Red Sea; therefore, it represents a unique case of a transition from rifting to spreading. The same conclusion follows from the analysis of detailed bathymetry structures and gravity observations^19,20^.

In this study, we present a new higher-resolution seismic tomographic model that shows some key lithospheric structures that were not observed in previous studies and that critically contribute to a better understanding of the geodynamics of the Red Sea area and lithospheric evolution during the rift to ridge transition.

## Data and tomographic inversion

Here, we jointly use the data of the Egyptian and Saudi Arabian networks containing the arrival times of the *P* and *S* waves from regional seismicity, which were never used together in tomography studies. In this research, we used a dataset with 15,899 regional and local events and correspondingly 111,981 *P*-wave and 20,157 *S*-wave arrival times (Figures S1 to S3 of Supplementary Materials). These data were inverted using the LOTOS code^21^. A detailed description of the data and the main algorithm workflow is presented in the Supplementary Materials.

The reliability of the model was carefully tested using a series of synthetic tests. We used both a checkerboard and a model with realistic anomalies similar to those obtained after inversion of the experimental data. The results of synthetic modeling and additional information can be found in the Supplementary Materials (Figures S4 to S7). Note that because of lower number data, the resolution of the *S*-wave model is much poorer. Therefore, for our interpretation, we mostly used the *P*-wave model.

**Figure 2:**
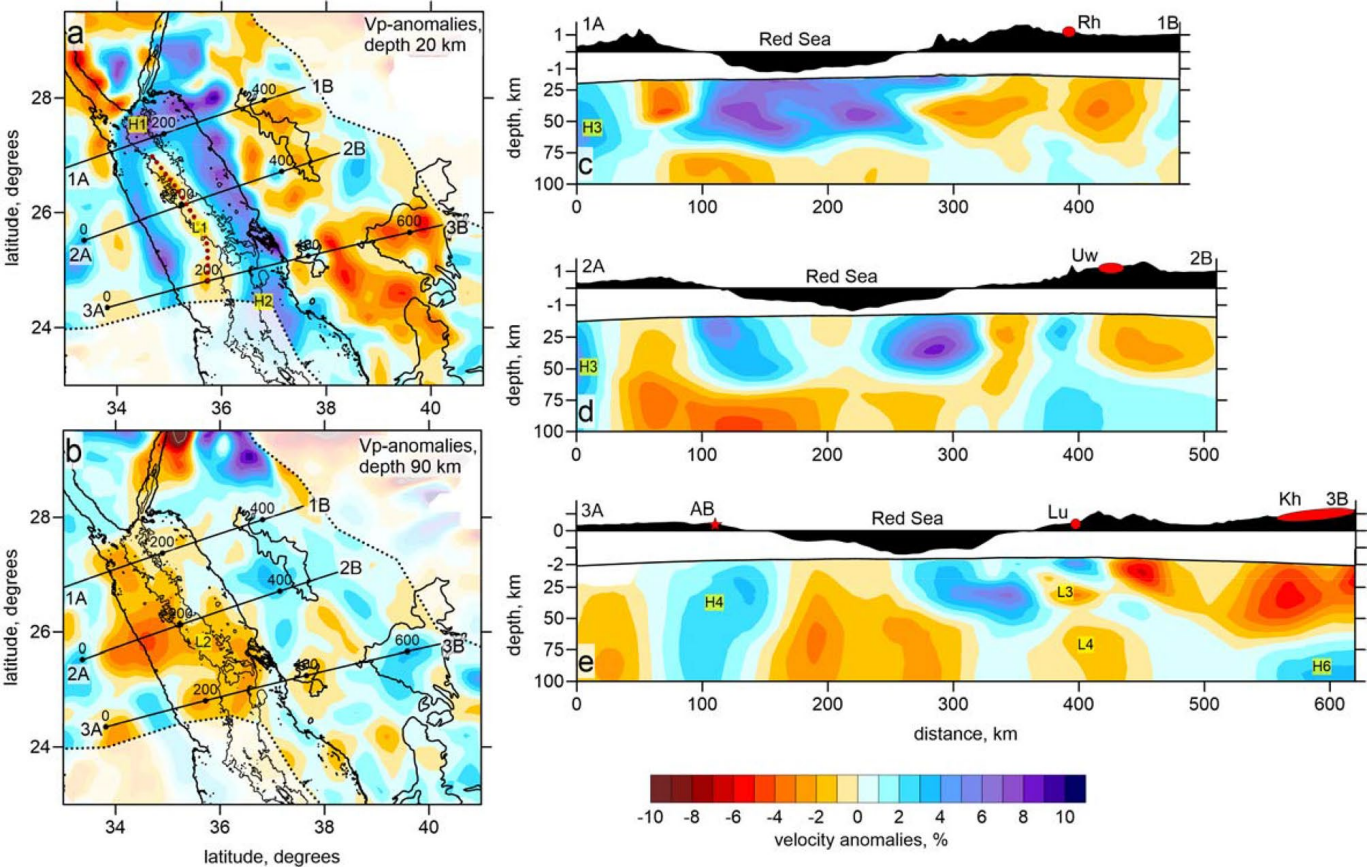
*P*-wave velocity anomalies obtained from tomographic inversion presented in two horizontal (panels **a**, **b**) and three vertical (panels **c**–**e**) sections. In the maps, the contours of harrats are shown on the Saudi Arabian side. Within the Red Sea, the bathymetry is shown with contour lines starting from 1000 m and at an interval of 500 m. Above each of the vertical sections, the topography/bathymetry along the profile is shown. The locations of the main harrats are shown in red ellipses as follows: Rh – ar Rahah, Uw – Uwayrid, Lu – Lunayyir, Kh – Khaybar, and Rt – Rahat. AB is Abu-Dabbab. Dotted lines indicate political boundaries. Letters in yellow indicate the structures discussed in the text. The images have been produced using the Surfer Golden Software 13 (https://www.goldensoftware.com/products/surfer).

The resulting distributions of the *P*-wave velocity anomalies of the experimental data inversion are shown in Fig. 2 in two horizontal sections and three vertical profiles. More horizontal sections of this model, as well as the results for the *S*-wave velocity model and absolute P-wave velocity in vertical sections, are presented in the Supplementary Materials (Figures S8 to S10). The final distribution of the seismicity after five iterations of the inversion is shown in Fig. 1B.

## Discussion

In the tomographic model, beneath the northern Red Sea (to the north of 24°N), we see a prominent high-velocity anomaly to 50–70 km depth whose shape approximately corresponds to the limits of the basin. A similar anomaly for the northernmost part of the Red Sea has already been retrieved by other tomographic studies merely based on the Egyptian networks^22,23^. The novel feature of the present result is an elongated low-velocity anomaly in the central part of the Red Sea between 24ºN and 27ºN (red dotted line with the indication of L1 in Fig. 2a). In the deeper sections, below 50 km depth, the negative anomaly beneath the Red Sea widens and is observed below the entire basin (anomaly L2 in Fig. 2b). At these depths, the high-velocity anomalies are traced as several distinct patterns mostly beneath onshore areas. In such regional-scale studies, areas of high seismic velocities are commonly associated with rigid, highly consolidated massifs consisting of igneous or deeply metamorphosed rocks, whereas the low-velocities are usually interpreted as sedimentary deposits or active magmatic systems with high temperature and/or melt content.

It is interesting that in areas where the low-velocity anomaly is observed beneath the axial trough, the seismicity is relatively weak whereas the maximum seismic activity is observed beneath the northernmost part of the Red Sea and at approximately 24ºN latitude at the vicinity of the high-velocity anomaly indicated H2. This irregularity in the seismicity distribution along the Red Sea axis was earlier discussed^24^. It was shown that high seismic activity corresponds to colder areas where the extending crust behaves in a brittle manner. This seems to be supported by the results of this study: a seismic gap between 25.5 °N and 26 °N and highly dispersed seismicity between 26 °N and 26.5 °N with earthquake magnitudes rarely exceeding 4 ^25^ correspond to low-velocity areas in the tomographic model along the rift axis (anomaly L1 in Fig. 2a), which may mark high-temperature zones where the ductile extension of the hot crust occurs. Alternatively, the extension in this segment may occur by dike intrusion rather than normal faulting, which could result in a net decrease in seismic moment release. Such mechanism was earlier proposed in the context the Afar extension^26–28^.

In another segment of the axial rift located in the southern part of our study area between the latitudes of 23ºN and 25ºN indicated with H2, the observed high velocity anomalies may characterize cold zones, where brittle crust deformation produces considerable seismicity.

**Figure 3:**
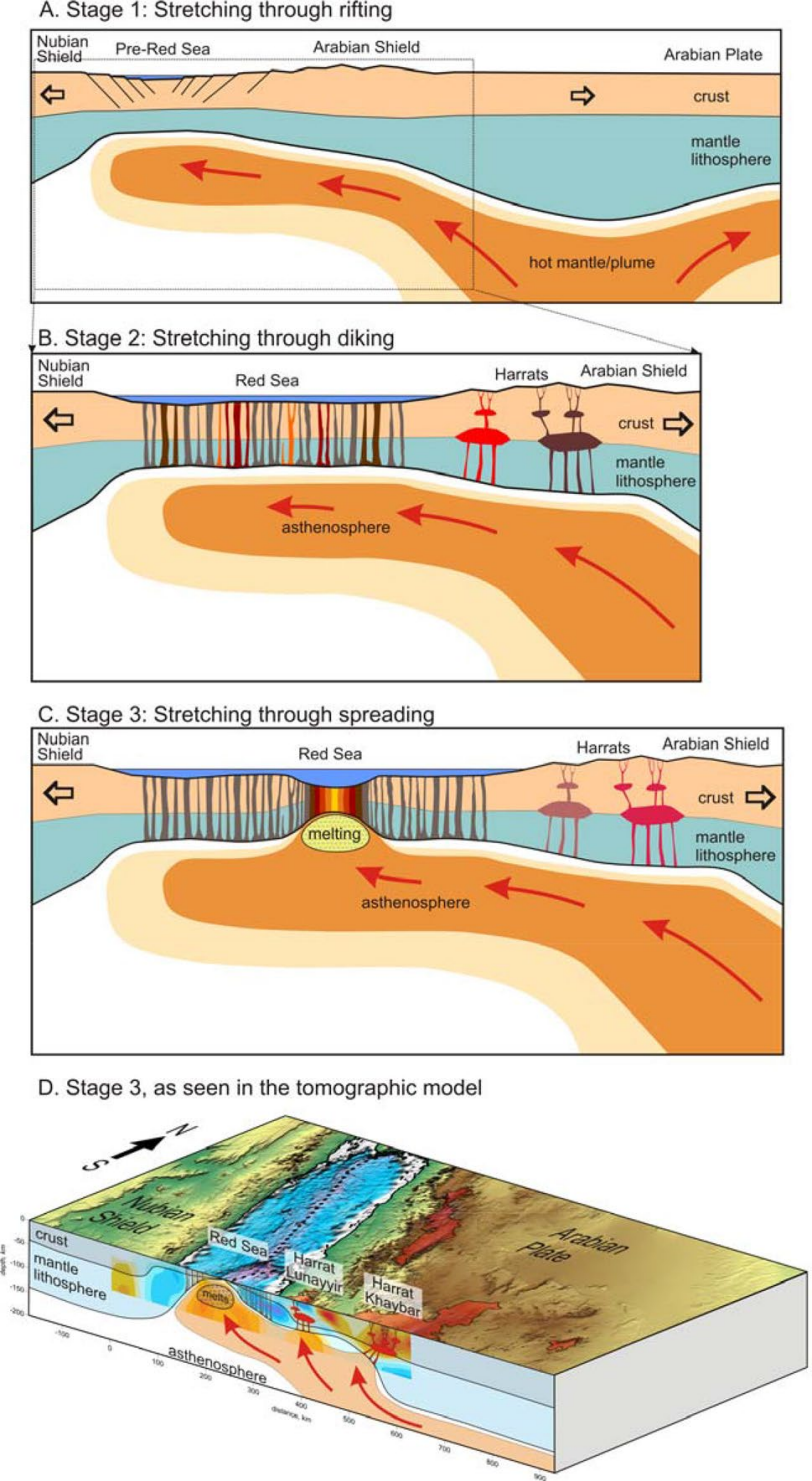
Schematic representation of three stages of extension of the transition from continental rifting to oceanic spreading (panels **A**–**C**). The interpretation in panel (**D**) is based on the results of seismic tomography obtained in this study. See details in the text. The images have been created in Corel Draw (coreldraw.com).

Based on the tomographic results, we propose that in the Red Sea area, we may observe gradual lithosphere reworking due to extension and initiation of the spreading zone, as schematically shown in Fig. 3. Before the opening of the Red Sea, between the divergent Arabian and Nubian plates, the lithosphere extension occurred through continuous stretching of continental crust (Stage 1 in Fig. 3A). The extension primarily occurred in a zone where the lithosphere was softened by the presence of hot asthenosphere that arrived either from the Afar Plume^29^ or from an overheated area beneath the central part of the Arabian Plate^30^. In this case, the continental crust was gradually thinned by normal faulting, as observed in other continental rifts.

In a case of the presence of hot asthenospheric material beneath extended lithosphere, as has been presumed beneath the western margin of the Arabian Plate, further extension of the continental lithosphere may trigger intrusions of the mantle-originated magma into the crust^31^ (Stage 2 in Fig. 3B). In this case, the lithosphere stretching due faulting and dike intrusion can occur synchronously, as supported by an evidence that extension was kick started by a phase of intrusions around ~ 25 Myrs ago^13^. Thick continental and thin transient lithosphere in this case behave differently. In the thicker lithosphere, as beneath the western margin of the Arabian Plate, the intrusions create a series of multi-level magma reservoirs that episodically lead to eruptions of massive basaltic fields, i.e. Harrats. For the extended thinner lithosphere, as within the Red Sea basin, the asthenospheric material creates a series of dikes propagating throughout the lithosphere that accommodate extension. Note that the emplacement of a dike releases local tensile crustal stresses making the respective crustal region not suitable for further fracturing. Therefore, the further extension of the lithosphere is accommodated by dikes emplacement in others more stressed parts of the lithosphere^32^. This explains why the dikes in the transient lithosphere uniformly affect the entire basin and are not localized in one narrow zone. After some time, these dikes composed of frozen igneous rocks change the effective integral properties of the lithosphere, making it fundamentally different from the stretched continental lithosphere. In the tomographic model of the current study (Fig. 2a), this transitional lithosphere appears as a high-velocity anomaly observed at shallow sections. We see that the coastal line of the Red Sea corresponds to a sharp transition from low velocity beneath the onshore areas to high velocity offshore without any gradual transition. This is particularly clear for the northern Red Sea area ("Introduction" section in Fig. 2) where the offshore lithosphere is uniformly high-velocity (anomaly H1 in Fig. 2a).

The third stage of the lithosphere stretching (Fig. 3C) presumes breaking of the lithosphere in a localized area and then further development of oceanic spreading. Possible interpretation of the derived distributions of the *P*-wave velocity anomalies in Sect. 3, which correspond to this extensional stage, is schematically shown in Fig. 3D. Spreading in the Red Sea is evidenced by the presence of the elongated low-velocity anomalies L1 (Fig. 2a) coinciding with the line of “deeps” along the axial part of the Red Sea. This low-velocity zone along the axial trough represents the hot partially molten asthenosphere that has ascended immediately below the spreading center. Note that the southernmost part of the anomaly L1 deviates westward from the through axis. This may indicate that the lithosphere fracturing in this zone avoids ductile high-temperature zones and primarily occurs in colder brittle lithosphere associated with high-velocity anomaly H2 and high seismicity.

The high-velocity anomalies along the opposite sides of the Red Sea basin represent the oceanic–continental transitional mantle lithosphere perturbed by a system of mafic dykes. These lithosphere segments appear to be not symmetrical. As we see in the vertical sections in Fig. 2, the lithosphere in the eastern flank of the Red Sea does not exceed 40 km in thickness, whereas beneath the Egyptian coast it appears much thicker. In the northern part of the Red Sea basin, the mantle lithosphere may be up to 50–70 km thick.

Based on the new tomography model, we can conclude that within the study area, a transition between two different mechanisms of rift basin opening exists: in the northern part, we propose an extension due to dispersed diking throughout the entire basin of the Red Sea (Scheme 2 in Fig. 3B), whereas in the southern part, most of extension is thought to be accommodated in a narrow zone of initiated spreading (Scheme 3 in Fig. 3C). This transition might be associated with a variable extension rate along the Red Sea, which is caused by the fact that the Arabian plate is rotating anticlockwise with an Euler pole somewhere in the Mediterranean Sea and this results in a decreasing spreading rate from the south to north of the Red Sea.

To understand tectono-magmatic processes leading to the formation of the transitional lithosphere, we analyzed the results of previously published numerical models of the rifting to spreading transition^3^, in which dynamics of magma addition to the lithosphere was simulated. The asymmetry of the lithospheric extension in the northern Red Sea area suggests the existence of rheologically decoupled continental lithosphere with a weak lower crust that greatly affects mantle melting and crustal growth dynamics^2,3^ (Fig. 6 in Liao and Gerya^3^). Compared to stronger and more symmetrical rheologically coupled models, decoupled lithosphere extension is characterized by a distinct transient period of enhanced mantle melting and related magmatic crustal growth. During this period, lithospheric extension can likely be dominated by magmatic (e.g., dike emplacement) rather than tectonic processes (a high-M regime, where M is the proportion of magmatic vs. tectonic extension^33^. As a result, a transitional, predominantly magmatic lithosphere can form as imaged in the high seismic velocity anomaly of the northern Red Sea area. During a later stage, localization of lithospheric extension produced an incipient oceanic ridge that disrupted the transitional lithosphere and formed a linear negative seismic velocity anomaly via localized upwelling and decompression melting of the upper mantle (Fig. 3C and D).

## Conclusions

We have constructed a 3D seismic model of the lithosphere and uppermost mantle beneath the Red Sea and surrounding areas based on a merged dataset containing *P*- and *S*-wave arrival times from regional seismicity provided by Egyptian and Saudi Arabian seismic networks. For the Red Sea basin, we have achieved a better resolution compared to the cases when the data of these networks were separately used. The derived seismic model resolves the lithospheric structure during the ongoing transition from continental breakup to oceanic spreading. In the Red Sea basin, particularly in its northernmost part, we observed a pronounced high-velocity anomaly. A sharp transition from low to high velocity along the Red Sea’s coastline may disprove the mechanism of gradual stretching of the continental crust during passive rifting. The results of this study may show that the crust in the northernmost Red Sea is principally different from the regular continental crust and represents a transient type of lithosphere strongly affected by dike intrusions. The linear low-velocity anomaly along the axial trough of the northern Red Sea shows a zone of spreading initiation causing asthenospheric ascent.

## Supplementary Information

Supplementary Information

## Data Availability

The full directory with data and program codes to reproduce the results presented in this paper can be downloaded from Zenodo. http://doi.org/10.5281/zenodo.4482096. This compressed file includes a Read_Me.pdf file with detailed guidelines on how to perform the calculations.
