# Author Correction: Transition from continental rifting to oceanic spreading in the northern Red Sea area

**DOI:** 10.1038/s41598-021-92049-7

**Published:** 2021-06-08

**Authors:** Sami El Khrepy, Ivan Koulakov, Taras Gerya, Nassir Al-Arifi, Mamdouh S. Alajmi, Ayman N. Qadrouh

**Affiliations:** 1 Natural hazards and mineral resources chair, Geology and Geophysics Department, King Saud University, P.O. Box 2455, Riyadh, 11451 Saudi Arabia; 2 Seismology Department, National Research Institute of Astronomy and Geophysics (NRIAG), Helwan, 11421 Egypt; 3 Trofimuk Institute of Petroleum Geology and Geophysics SB RAS, Prospekt Koptyuga, 3, Novosibirsk, Russia 630090; 4 Novosibirsk State University, Pirogova 2, Novosibirsk, Russia 630090; 5 Institute of Volcanology and Seismology FEB RAS, Piip Boulevard, 9, Petropavlovsk-Kamchatsky, Russia 693006; 6 Department of Earth Sciences, ETH Zurich, Sonneggstrasse 5, 8092 Zurich, Switzerland; 7 King Abdulaziz City of Science and Technology, Riyadh, Saudi Arabia

Correction to: *Scientific Reports* 10.1038/s41598-021-84952-w, published online 10 March 2021

The original version of this Article contained an error in Affiliation 1, which was incorrectly given as ‘Natural Hazards and Mineral Resources, Geology and Geophysics Department, King Saud University, P.O. Box 2455, Riyadh 11451, Saudi Arabia’.

The correct affiliation is listed below:

Natural hazards and mineral resources chair, Geology and Geophysics Department, King Saud University, P.O. Box 2455, Riyadh 11451, Saudi Arabia.

The original Article and accompanying Supplementary Information file have been corrected.

