# Supplementary material for: Transition from continental rifting to oceanic spreading in the northern Red Sea area
Source: Sci Rep. 2021 Mar 10;11:5594. doi: 10.1038/s41598-021-84952-w (PMC7946956; doi:10.1038/s41598-021-84952-w)
Supplement: Supplementary file 1 — Supplementary Information [file 41598_2021_84952_MOESM1_ESM.docx]

Supplementary materials to the paper:

**Transition from continental rifting to oceanic spreading in the northern Red Sea area**

Sami El Khrepy^1,2^, Ivan Koulakov^3,4,5^, Taras Gerya^6^, Nassir Al-Arifi^1^,
Mamdouh S. Alajmi^7^ and Ayman N. Qadrouh^7^

*^1^ Natural hazards and mineral resources chair, Geology and Geophysics Department, King Saud University, P.O. Box 2455, Riyadh 11451, Saudi Arabia (* [*k_sami11@yahoo.com*](mailto:k_sami11@yahoo.com)*,* [*nalarifi@ksu.edu.sa*](mailto:nalarifi@ksu.edu.sa))

*^2^ National Research Institute of Astronomy and Geophysics, Seismology Department, NRIAG, Helwan 11421, Egypt.*

*^3^ Trofimuk Institute of Petroleum Geology and Geophysics, SB RAS, Prospekt Koptyuga, 3, Novosibirsk 630090, Russia.*

*^4^ Novosibirsk State University, Novosibirsk, Russia, Pirogova 2, Novosibirsk 630090, Russia.*

*^5^ Institute of Volcanology and Seismology FEB RAS, Piip Boulevard, 9, 693006, Petropavlovsk-Kamchatsky, Russia*

*^6^ ETH Zurich, Dep. Of Earth Sciences, Sonneggstrasse 5, Zurich 8092, Switzerland.*

*^7^ King Abdulaziz City of Science and Technology, Riyadh, Saudi Arabia (malajmi@kacst.edu.sa, aqadrouh@kacst.edu.sa).*

In these supplementary materials we provide details on previous studies of the Red Sea area and its surroundings, discuss some technical issues related to the performance of the tomography procedure, and give some information on synthetic modeling.

**Previous studies of the Red Sea and its surroundings**

Understanding the mechanisms of rifting and volcanism in a region requires robust and reliable information about its crustal and mantle structures. In the area of the Red Sea, a number of different geophysical studies have been performed over more than 50 years. An overview of the various geophysical and geological investigations into the Red Sea area is given by (7) and in a monograph by (6). The magnetic measurements were mainly used to discover linear magnetic anomalies, indicating the spreading process (8, 34), and to identify fault systems as well (35). The gravity observations reveal a linear positive high-density anomaly associated with thin crust, which is especially clear in the south and looks more dispersed in the northern part of the Red Sea (19,20,36). The heat flow is elevated throughout the Red Sea basin and is especially high along the axial trough, reaching 467 mW m^-2^ in its southern part and 250 mW m^-2^ to the north (37).

The lithosphere thickness in the adjacent areas of Saudi Arabia unperturbed by the extension is estimated to be ~140 km (38) and is expected to be similar in the Egyptian side. In the rift zone, the lithosphere is considerably thinner; (38) report a thickness of 40–60 km near the Red Sea, but in the extensional basin it might appear to be thinner. Based on seismic refraction data (39) the crustal thickness in central parts of Saudi Arabia may exceed 40 km, and the crust becomes 15–20 km thick at its coastal margins (40).

Most of the seismicity in the Red Sea occurs in the northern part of the basin including the Gulf of Suez, Gulf of Aqaba, and the southern Sinai tip, where the intersection of the plate boundaries (Red Sea and Dead Sea rifts) takes place. Along the rift axis of the Red Sea, the seismicity is distributed non-uniformly, something that may indicate variable thermal regimes determining ductile and brittle types of extension (24). In the adjacent onshore areas of Saudi Arabia and Egypt, the seismicity occurs in broad areas at distances of several hundred kilometers from the Red Sea. In many cases, the earthquakes do not form distinct clusters and apparently are not associated with a distinguishable fault system (Figure 1b). In western Saudi Arabia, some of the earthquake sequences are associated with magmatic activity beneath the harrats (41).

The Red Sea and adjacent areas of Saudi Arabia have been studied by a large number of active seismic source offshore and onshore experiments, which have provided information about their detailed structure (42, 43). However, these studies relate to specific locations of the Red Sea and cannot be easily interpolated to build a complete image of the Red Sea deep interior.

The crustal and upper mantle structures beneath the onshore area of Saudi Arabia have been investigated in a number of seismological studies. The regional mantle structures were studied by (44, 45, 30) based on regional and global travel time data. The data on the regional seismological network of Saudi Arabia were used to perform surface wave tomography (29, 46, 47, 48), receiver function analysis (49, 50), joint surface wave and receiver function (51, 52, 53), anisotropy from SKS splitting (54, 55), and teleseismic tomography (56, 57). In particular, the teleseismic surface-wave tomography has been performed based on stations located on the both sides of the southern Red Sea region (58, 59). Subsets of these data were used to study some local sites, such as Harrat Lunayyir (60) and Harrat Al-Madinah (61). As far as we aware, these data were never used to perform travel time tomography for the whole region.

In the Egyptian part, the investigations of deep structures were mainly based on travel time tomography with the use of the data from the national Egyptian network supplemented with some data from the ISC from the surrounding countries. Previously, these data were used to investigate the deep structures beneath the Gulf of Suez (22) and the Gulf of Aqaba (23). These models partly covered the area of the Northern Red Sea and revealed a strongly positive crustal velocity anomaly located beneath the offshore areas, which was interpreted as the result of a strongly extended crust saturated with magmatic dikes. A joint inversion of gravity and seismic tomography data of Egypt revealed a distinct lithosphere fragmentation of Egypt and asymmetric lithosphere structure of the northern Red Sea strongly related to its seismicity pattern (25).

Joint use of travel time data from the Egyptian and Saudi Arabian seismological networks gives much better ray coverage for the Red Sea basin than that in cases of separate inversions. As far as we know, these data have never been used in combination, and the present work provides the first 3D (three-dimensional) image of the Red Sea and surrounding areas of Egypt and Saudi Arabia based on passive travel time tomography. In this study, we found new features that help us make progress in solving the geodynamic problems mentioned above.

**Data and algorithms**

In this study, we used data from the Egyptian and Saudi Arabian seismic networks (Figure S1). The Egyptian dataset was supplemented by the ISC data from stations located in surrounding countries including Saudi Arabia. In total, in the Egyptian dataset, we used 93,588 *P*-waves and 23,112 *S*- wave arrival times from 16,623 regional events. The Saudi Arabian dataset included the 287,474 *P*- and 7,081 *S*-wave arrival times from 47,330 local, regional, and teleseismic events.

When selecting the events for tomography, we determined a circle with a radius of 700 km centered at 37ºE and 24ºN. All events outside this circle were not considered. Further data selection was performed during the source location in the starting 1D (one-dimensional) model. We used two criteria: (1) the number of picks per event should not be smaller than 8 and (2) the residual after location in the 1D model should not exceed 2 s of absolute value. As a result, for tomography, we selected 15,899 events with 111,981 *P* and 20,157 *S*-wave picks (8.3 picks per event).

To start the tomography inversion, we used a 1D reference model, in which the P and S wave velocities were defined at several depth levels and linearly interpolated in between. The values of the reference velocities were defined after several runs of the entire tomographic procedure: after each run, we calculated the average velocity values at corresponding depth levels and then used them to define the reference model for the next run. The velocities in the final reference model are presented in Table S1.

| Table S1. Reference 1D velocity distribution of the P and S wave velocities used to calculate the main tomography model | | |
| --- | --- | --- |
| Depth, km | Vp, km/s | Vs, km/s |
| -5 | 5.00 | 2.87 |
| 20 | 6.58 | 3.78 |
| 40 | 7.57 | 4.35 |
| 60 | 7.98 | 4.58 |
| 100 | 8.05 | 4.62 |

In this study, we used the passive source tomography algorithm LOTOS (21), which performs simultaneous inversion for the P and S wave velocity and source parameters. The calculation procedure started from the preliminary source location, which used a stable grid-search method that does not depend on the starting search point. The travel times at this stage were calculated based on a simplified method using linear interpolation of tabulated values calculated in a preliminary step. At this step, we also performed the data selection according to the criteria considered in the previous paragraph.

The sources were then relocated using the more sophisticated program based on the gradient descending method and 3D ray tracing (bending algorithm with the basic principles proposed by (62). Every new iteration began with this relocation procedure based on a newly updated 3D velocity model.

The velocity models were parameterized using a set of nodes installed in the study area according to the ray coverage. In map view, the spacing between nodes was constant (25 km in our case), but they were placed only in areas with sufficient ray density (0.1 of the average value). In the vertical direction, the spacing inversely depends on the ray density, but it cannot be smaller than a predefined minimum value (10 km in our case). To avoid any dependency on the grid geometry, we performed independent inversions in four grids with different basic orientations (0º, 22º, 45º, and 66º) and then averaged the resulting models. The grid was constructed in the first iteration only; then the results were updated in the same nodes.

The inversion was performed simultaneously for the *P* and *S* wave velocity anomalies, and for the source parameters (*dx*, *dy*, *dz*, and *dt_0_*). The algorithm also allows inversion for station corrections, but this option was not used in our case. To stabilize the inversion, we used two types of regularization: amplitude damping and flattening. In our case, the amplitude damping parameters were equal to 0.4 and 1, for the *P* and *S* wave models respectively. The corresponding flattening coefficients were 2.5 and 6. The inversion of the matrix was performed using the LSQR method (63, 64).

The iterative procedure included the source re-location in the updated 3D velocity models, first derivative matric calculation, and inversion. In total, we performed five iterations. This number, as well as values of most inversion parameters, was determined based on synthetic modeling. In Figures S2 and S3, we show the final distributions of the ray paths and events after five iterations of inversions and source relocations. Although most of seismicity is located above 30 km depth, many refracted rays at large epicentral distances dive down to 60 km and even deeper, which enable fair resolution of the tomography model in the uppermost mantle.

**Synthetic modeling**

Synthetic modeling is an important stage that allows for adequate estimates of the resolution capacity and reliability of the derived models. Furthermore, it can be used to tune the optimal values of the main controlling parameters that enable the best possible quality of the recovered model. The synthetic model is defined as a superposition of the reference 1D model and the 3D distribution of synthetic anomalies. Here, we present two types of models: checkerboard (Figure S4), and anomalies with realistic shapes (Figure S5). The synthetic travel times were calculated using the 3D bending ray tracer for the existing source-receiver pairs taken from the experimental model in a final iteration. After calculation of the synthetic data, we “forgot” about the source coordinates and the origin times and performed the recovery procedure using an identical workflow and controlling parameters as in the case of experimental data analysis. In particular, the first step of absolute source locations led to considerable shifts of sources in respect to their true locations, which adequately simulates the problem of source location uncertainty due to the trade-off between source and velocity parameters. This is especially important when outside network events are involved in the calculation, for which we could not provide accurate locations.

In Figure S4, we show the result of recovery of the checkerboard model consisting of the alternating anomalies with the amplitudes of ±7% and lateral size of 100 km. In this case, the anomalies did not change with depth. In the recovery results, the *P*-wave velocity anomalies are correctly recovered in most parts of the study area; however, within the Red Sea, the anomalies are smeared and not always visible. For the velocity of the *S*-waves, this test fails almost completely. With the available amount of *S*-wave data, we are not able to resolve features of 100 km size.

Figure S5 presents more realistic model, which includes the high-velocity anomaly coinciding with the Red Sea basin, narrow low-velocity anomaly along the rift axis and several negative anomalies associated with harrats in the western margin of the Arabian Plate. The recovery results for the *P*-wave velocity look very similar to the results of the experimental data inversion. The high-velocity anomaly in the basin of the Red Sea is generally well recovered. However, we see that at shallow depths, we cannot recover continuously the axis-related low-velocity anomaly. We can observe it in the northern and southern segments, but at around 24ºN latitude, it is interrupted by a strong high-velocity anomaly. At greater depths, this anomaly grows more similar in shape to the original. The harrat-related anomalies in the western margin of the Arabian Plate are recovered correctly. It is important that for the *S*-wave velocity model, the main patterns, except for the rift-related anomaly, are recovered correctly despite a lower number of data.

The next series of tests shown in Figure S6 aim at checking the vertical resolution. Because of the fundamental problem of the trade-off between the velocity and source parameters (especially for the source depth) the resolution in the passive source tomography is usually poorer than the vertical resolution. Therefore, we present several tests showing the capacity of the tomographic inversion to resolve the change of the anomaly sign at 30 km depth, similarly to what we observe in the main model based on the experimental data. The synthetic model is defined along each of five vertical profiles used for presenting the main results. We define the checkerboard with 100 km spacing along the section and 60 km spacing with depth (anomaly changes at 30 km, 90 km and so on). Across the section, the anomalies have a thickness of 200 km. We can see that the sign transition at 30 km depth is robustly resolved in most of the sections. Poorer vertical resolution is obtained in the offshore areas beneath the Red Sea. The *S*-wave data do not allow us to recover any change with depth.

The synthetic tests allow us estimating the uncertainty of source locations. It should be noted that in the case of experimental data, the depths of events were larger than expected, and it was likely an artifact caused by poor station coverage. In Figure S7, we present the result of source location after five iterations corresponding to the checkerboard test shown in Figure S2. We should remind that in our testing workflow, the travel times are calculated through the 3D synthetic model; then we “forget” the coordinates and origin times of the sources and locate them using a starting 1D model. Because of a considerable difference between the true and starting models, the sources appear to be strongly biased with respect to the true locations. After several iterations of the velocity recovery, the sources get closer to their true locations, but if the station coverage is not good, they remain strongly mislocated. In Figure S7, we see that the inversion cannot ensure the accurate locations of sources. For some events the errors may reach to dozens of kilometers, and the mean mislocation value is equal to 5.22 km. In this test, we can see that the inversion led to considerable deepening of some events, and we can expect the similar bias in the case of experimental data inversion. At the same time, these source location errors do not prevent obtaining reasonable horizontal resolution in checkerboard tests, as seen in Figures S4 and S5, and we expect that similarly robust images can be derived from the experimental data inversion. We cannot accurately determine the source coordinates, because solutions at two different locations at two sides of the bars in Figure S7 provide approximately same values of time residuals. This gives us a possibility to recover the velocity anomalies in the tomographic inversion, despite poor determination of the sources. In this case, in the velocity model, we may lose some vertical resolution, but the horizontal resolution remains fair.

In summary, the synthetic tests show limited resolution both in the horizontal and vertical directions. This should be taken into account when interpreting the models. Nevertheless, these synthetic tests have demonstrated that anomalies similar in size to the major structures, which are discussed in the main paper, can be robustly resolved by the synthetic models and, therefore, are reliable.

**Experimental data inversion**

The main model resulted from the inversion of experimental data was obtained after five iterative steps of the tomographic inversion and source relocation. The values of the residuals and variance reduction (both in the L1 norm) for this model are shown in Table S2. It can be seen that for both *P* and *S* wave velocity model, the variance reduction is around 30%.

In addition to the P-wave velocity model shown in Figure 2 of the main paper, in the supplementary Figures S8 to S9, we show the *P* and *S* wave velocity models in more horizontal sections. Furthermore, in Figure S10, we show the absolute values of the P-wave velocities in three vertical sections, same as used for showing the main results in Figure 2 of the main paper. Actually, seismic tomography provides continuous distributions of seismic velocity without any first-order contrasts. At the same time, these results may be helpful to reveal deviations of some interfaces with strong velocity contrasts, such as Moho. In this case, the thicker crust is associated with low-velocity anomalies at the corresponding depths, whereas the thinner crust corresponds to higher velocity. Correspondingly, contour lines in the absolute velocity model in vertical sections may represent deviations of the major interfaces. For example, the iso-velocity at ~7.3 km/s (violet layer) in Figure S10 may correspond to Moho, which is shallower beneath the Red Sea basin and deeper beneath surrounding continental areas. At the same time, we should keep in mind that during the inversion we use damping that help us to stabilize the solution and reduce the effect of noise in the data, but may smear the resulting anomalies and reduce their amplitudes. As a result, the interface variations estimated from the shapes of absolute velocity contour lines may appear to be strongly underestimated and we cannot use them for accurate determination of crustal thickness. For this and other reasons, we find maps with relative anomalies (Figure 2 of the main paper) much more informative, as they reveal many details, which are not visible in absolute velocity images. Thus, we consider them as the main result of this study and primarily use them for our discussion.

Figure S10 also shows the distributions of events in the vicinity of the profiles. It should be noted that the depth determination, especially in the Red Sea area, cannot be ensured with the existing sparse station coverage. At the same time, this uncertainty also existed in the cases of synthetic modeling, in which the locations of sources were “forgotten”. In Figure S7, we see that the sources tend to be deeper than in the original model. In the case of experimental data inversion, the depths of the sources appear to be much larger than expected, and this might be an artifact caused by poor station coverage. At the same time, the synthetic tests have also demonstrated that large mislocations of sources did not prevent obtaining fair images of velocity variations. Therefore, we expect that the source depth mislocations do not affect dramatically the velocity model in the case of the experimental data inversion.

The geodynamic interpretation of this model is given in the main paper.

| **Table S2.** Values of the *P* and *S* wave residuals and their reduction during the iterative tomographic inversion of the experimental data | | | | |
| --- | --- | --- | --- | --- |
| iteration | dt *P*, s | reduction, dt *P*, % | dt *S*, s | reduction, dt *S*, % |
| 1 | 0.262 | 0.00 | 0.349 | 0.00 |
| 2 | 0.213 | 18.96 | 0.276 | 20.74 |
| 3 | 0.199 | 24.11 | 0.256 | 26.61 |
| 4 | 0.193 | 26.24 | 0.247 | 29.10 |
| 5 | 0.189 | 27.81 | 0.241 | 30.71 |

**References for the supplementary materials:**

1. Röser, H.A. A detailed magnetic survey of the southern Red Sea. *Geologie Jahrbuch* 13,131–153 (1975).
2. Hall, S.A. Magnetic evidence for the nature of the crust beneath the southern Red Sea. *J. Geophys. Res*. **94**, 12267–12279 (1989).
3. Izzeldin, Y.A. Seismic, gravity and magnetic surveys in the central part of the Red Sea; their interpretation and implications for the structure and evolution of the Red Sea. *Tectonophysics* **143**, 269–306 (1987).
4. Anschutz, P., Blanc, G., Chatin, F., Geiller, M. & Pierret, M-C. Hydrographic changes during 20 years in the brine-filled basins of the Red Sea. *Deep Sea Res Part 1 Oceanogr. Res. Pap.* **46**, 1779–1792 (1999).
5. Hansen, S.E., Rodgers, A.J., Schwartz, S.Y. & Al-Amri, A.M. Imaging ruptured lithosphere beneath the Red Sea and Arabian Peninsula. *Earth Planet. Sci. Lett.* **259***,*(3-4), 256-265 (2007).
6. Gettings, M.E., Blank, H.R., Mooney, W.D. & Healy, J.H. Crustal structure of southwestern Saudi Arabia. *J. Geophys. Res.* **91,** 6491– 6512 (1986).
7. Makris, J. *et al*. Crustal structure in the northwestern region of the Arabian shield and its transition to the Red Sea. *King Abdulaziz Univ. Bull. Fac. Earth Sci.* **6**, 435–447 (1983).
8. Pallister, J.S. *et al.* Broad accommodation of rift-related extension recorded by dyke intrusion in Saudi Arabia. *Nature Geoscience* **3**, 705–712 (2010).
9. Milkereit, B. & Flüh, E.R., Saudi Arabian refraction profile: crustal structure of the Red Sea-Arabian shield transition. *Tectonophysics* **111**,(3-4), 283-298 (1985).
10. Rihm, R., Makris, J. & Möller, L., Seismic surveys in the northern Red Sea: asymmetric crustal structure. *Tectonophysics* **198**,(2-4), 279-295 (1991).
11. Nyblade, A., Park, Y. Rodgers A. & Al-Amri, A. Seismic structure of the Arabian Shield lithosphere and Red Sea margin, *Margins Newsl.* **17**, 13–15 (2006).
12. Park, Y., Nyblade, A. Rodgers, A. & Al-Amri, A. Upper mantle structure beneath the Arabian Peninsula from regional body wave tomography: Implications for the origin of Cenozoic uplift and volcanism in the Arabian Shield. *Geochem. Geophys. Geosyst.* **8**, Q6021, doi:10.1029/2006GC001566. (2007).
13. Park, Y., Nyblade, A.A. Rodgers, A.J., & Al‐Amri, A. S wave velocity structure of the Arabian Shield upper mantle from Rayleigh wave tomography. *Geochem. Geophys. Geosyst.* **9**, (7) (2008).
14. Yao, Z., Mooney, W.D., Zahran, H.M. & Youssef, S.E.H. Upper mantle velocity structure beneath the Arabian shield from Rayleigh surface wave tomography and its implications. *J. Geophys. Res.: Solid Earth* **122** (8), 6552-6568 (2017).
15. Tang, Z., Mai, P.M., Chang, S.J. & Zahran, H. Evidence for crustal low shear-wave speed in western Saudi Arabia from multi-scale fundamental-mode Rayleigh-wave group-velocity tomography. *Earth Planet. Sci. Lett.* **495**, 24-37 (2018).
16. Al-Damegh, K., Sandvol, E. & Barazangi, M. Crustal structure of the Arabian plate: New constraints from the analysis of teleseismic receiver functions. *Earth Planet. Sci. Lett.* **231**(3), 177–196, doi:10.1016/j.epsl.2004.12.020 (2005).
17. Hansen, S.E., Rodgers, A.J., Schwartz, S.Y. & Al-Amri, A.M. Imaging ruptured lithosphere beneath the Red Sea and Arabian Peninsula. *Earth Planet. Sci. Lett.* **259***,*(3-4), 256-265 (2007).
18. Julià, J., Ammon, C. J. & Herrmann, R. B. Lithospheric structure of the Arabian Shield from the joint inversion of receiver functions and surface-wave group velocities. *Tectonophysics* **371**(1), 1–21, doi:10.1016/S0040-1951(03)00196-3 (2003).
19. Tkalčić, H. *et al.* A multistep approach for joint modeling of surface wave dispersion and teleseismic receiver functions: Implications for lithospheric structure of the Arabian Peninsula, *J. Geophys. Res.* **111**, B11311, doi:10.1029/2005JB004130 (2006).
20. Mai, P.M., Julià, J. & Tang, Z. Crustal and upper-mantle structure beneath Saudi Arabia from receiver functions and surface wave analysis. *Geological Setting, Palaeoenvironment and Archaeology of the Red Sea* (pp. 307-322). Springer, Cham. (2019).
21. Elsheikh, A. A., Gao, S. S., Liu, K. H., Mohamed, A. A., Yu, Y. & Helbary, R. E. Fat. Seismic anisotropy and subduction-induced mantle fabrics beneath the Arabian and Nubian Plates adjacent to the Red Sea. *Geophys. Res. Lett.* **41**, 2376–2381, doi:10.1002/2014GL059536 (2014).
22. Wolfe, C. J., Vernon, F. L. & Al-Amri, A. Shear-wave splitting across western Saudi Arabia: The pattern of uppermantle anisotropy at a Proterozoic shield, *Geophys. Res. Lett.* **26**(6), 779–782, doi:10.1029/1999GL900056 (1999).
23. Benoit, M. H., Nyblade, A. A., VanDecar, J. C. & Gurrola, H. Upper mantle P wave velocity structure and transition zone thickness beneath the Arabian Shield, *Geophys. Res. Lett.* **30**(10), 1531, doi:10.1029/2002GL016436 (2003).
24. Korostelev, F. *et al.* Crustal and upper mantle structure beneath south-western margin of the Arabian Peninsula from teleseismic tomography, *Geochem. Geophys. Geosyst*. **15**, 2850–2864, doi:10.1002/2014GC005316 (2014).
25. Gallacher, R. *et al*. The initiation of segmented buoyancy-driven melting during continental breakup. *Nature Communications*, 7, 13110, https://doi.org/10.1038/ncomms13110 (2016)
26. Gallacher, R., Keir, D., & Harmon, N., The nature of upper mantle upwelling during initiation of seafloor spreading in the southern Red Sea. In *Geological Setting, Palaeoenvironment and Archaeology of the Red Sea*, 113-129, <https://doi.org/10.1007/978-3-319-99408-6_6> (Springer, Cham. 2019)
27. Koulakov, I., El Khrepy, S., Al-Arifi, N., Kuznetsov, P. & Kasatkina, E. A. Structural cause of a missed eruption in the Harrat Lunayyir basaltic field (Saudi Arabia) in 2009. *Geology* **43**(5), 395-398 (2015).
28. Abdelwahed, M.F. *et al.* Imaging of magma intrusions beneath Harrat Al-Madinah in Saudi Arabia. *J. Asian Earth Sci.* **120**, 17-28 (2016).
29. Um, J. & Thurber, C.H. A fast algorithm for two-point seismic ray tracing. *Bull. Seismol. Soc. Am*. **77**, 972–986 (1987).
30. Paige, C.C. & Saunders, M.A. LSQR: An algorithm for sparse linear equations and sparse least squares. *ACM Transactions on Mathematical Software* **8**, 43–71 (1982).
31. Nolet, G. Seismic wave propagation and seismic tomography. *Seismic Tomography. Edited by G. Nolet,* pp 1–23, Reidel, Dordrecht (1987).


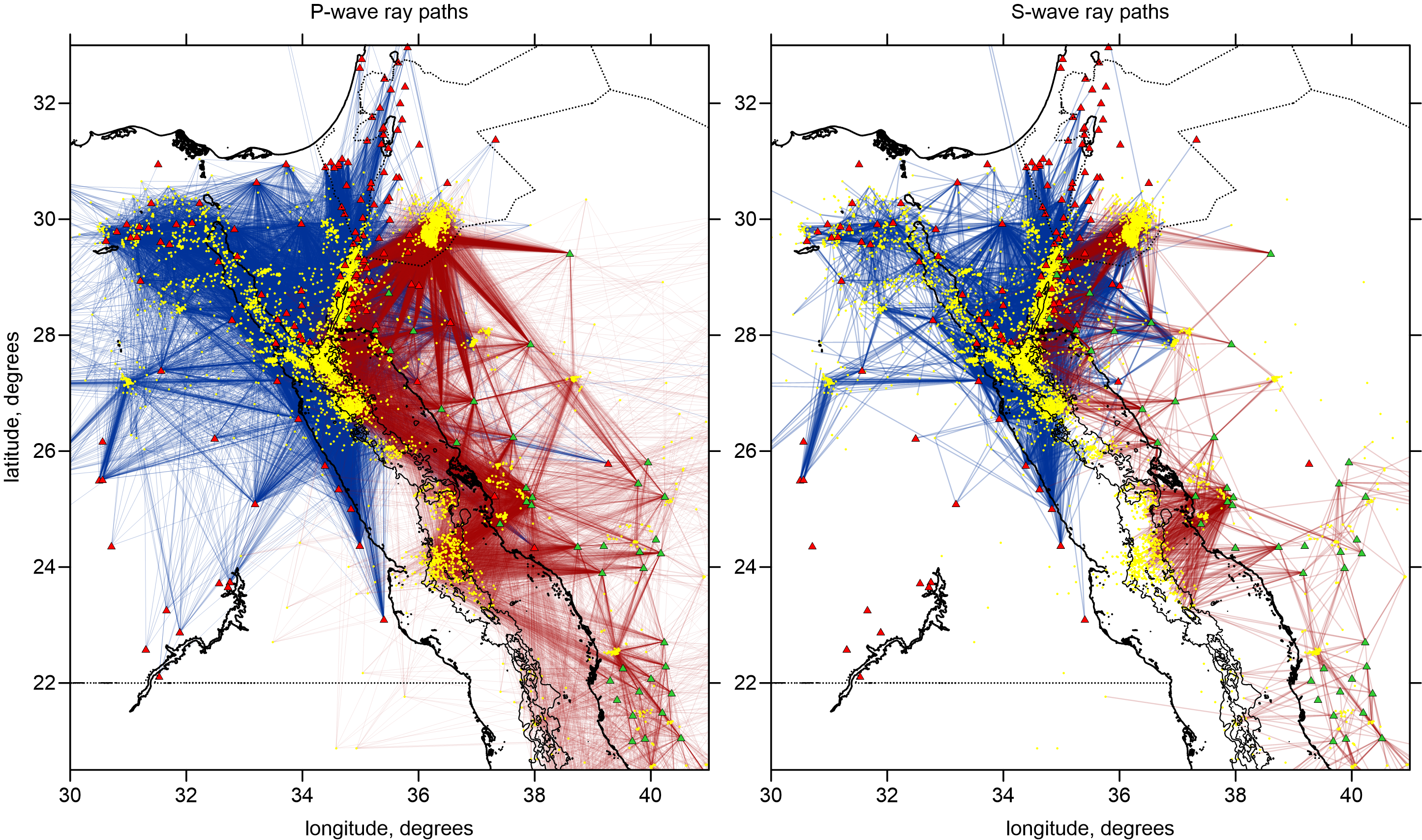


Figure S1. Distributions of the P- and S-wave ray paths corresponding to the Egyptian (blue) and Saudi Arabian (red) data subsets. Involved stations of the Egyptian network supplemented with the ISC stations are depicted with red triangles; stations of the Saudi Arabian network are shown with green triangles. The yellow dots depict the seismicity used in this study. Dotted lines indicate political boundaries. The images have been produced using the Surfer Golden Software 13 (https://www.goldensoftware.com/products/surfer)


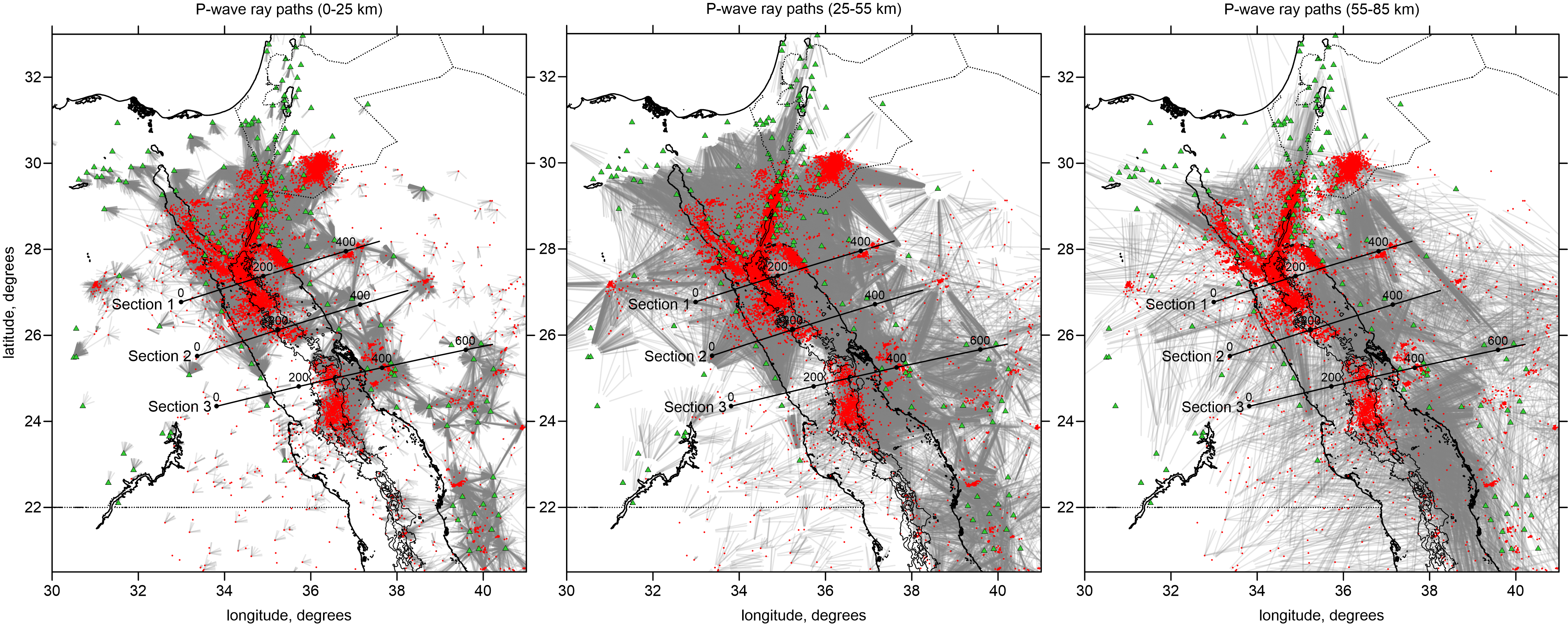


Figure S2. Distributions of the P-wave ray paths (gray lines) in three depth intervals. Red dots depict the events used for tomography; green triangles indicate seismic stations. Dotted lines indicate political boundaries. The images have been produced using the Surfer Golden Software 13 (https://www.goldensoftware.com/products/surfer)


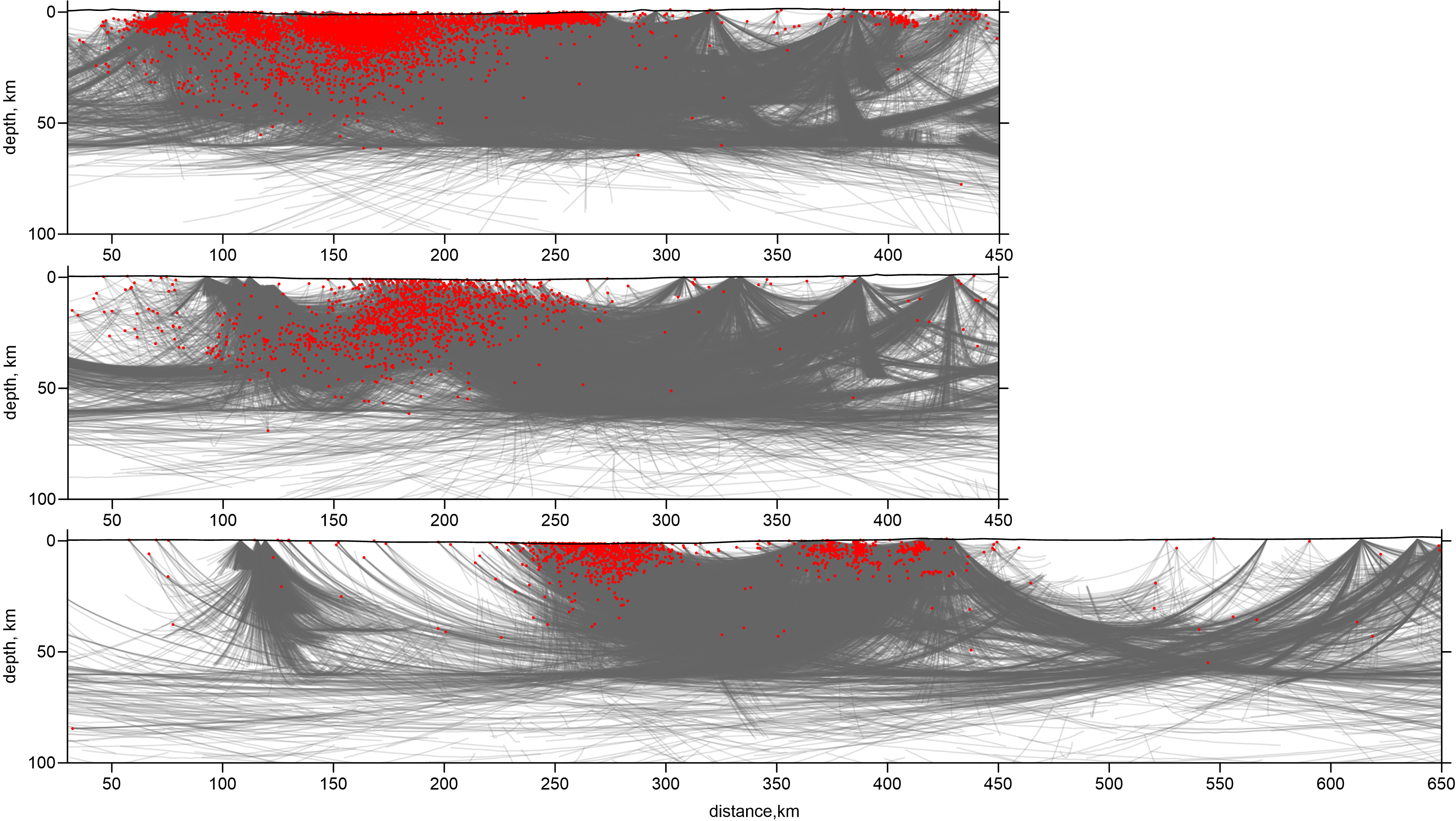


Figure S3. Distributions of the P-wave ray paths (gray lines) along three vertical sections indicated in Figure S2. Red dots depict the events used for tomography. Both rays and events are within the distance of 100 km from the profile. The images have been produced using the Surfer Golden Software 13 (https://www.goldensoftware.com/products/surfer)


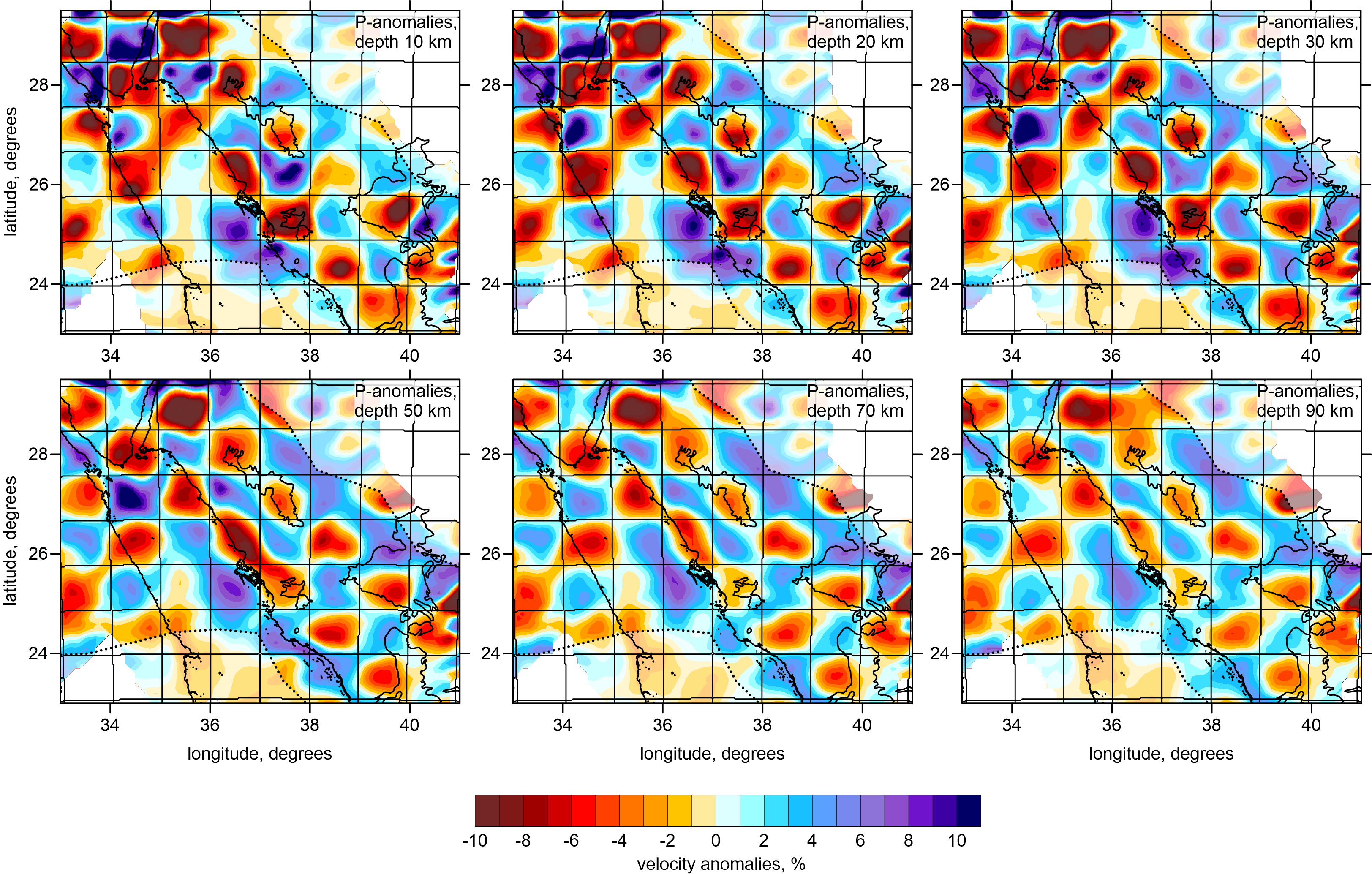


Figure S4. Checkerboard test for the P –wave velocity anomalies. Size of anomalies is 100 km. Initial synthetic anomalies are shown with black contour lines. Note that for the most parts of the onshore areas of Egypt and Saudi Arabia, as well as for the northern Red Sea, the anomalies are fairly restored. South the 25 N latitude, beneath the onshore areas of the Red Sea, the resolution is lower. Dotted lines indicate the resolved area. The images have been produced using the Surfer Golden Software 13 (https://www.goldensoftware.com/products/surfer)


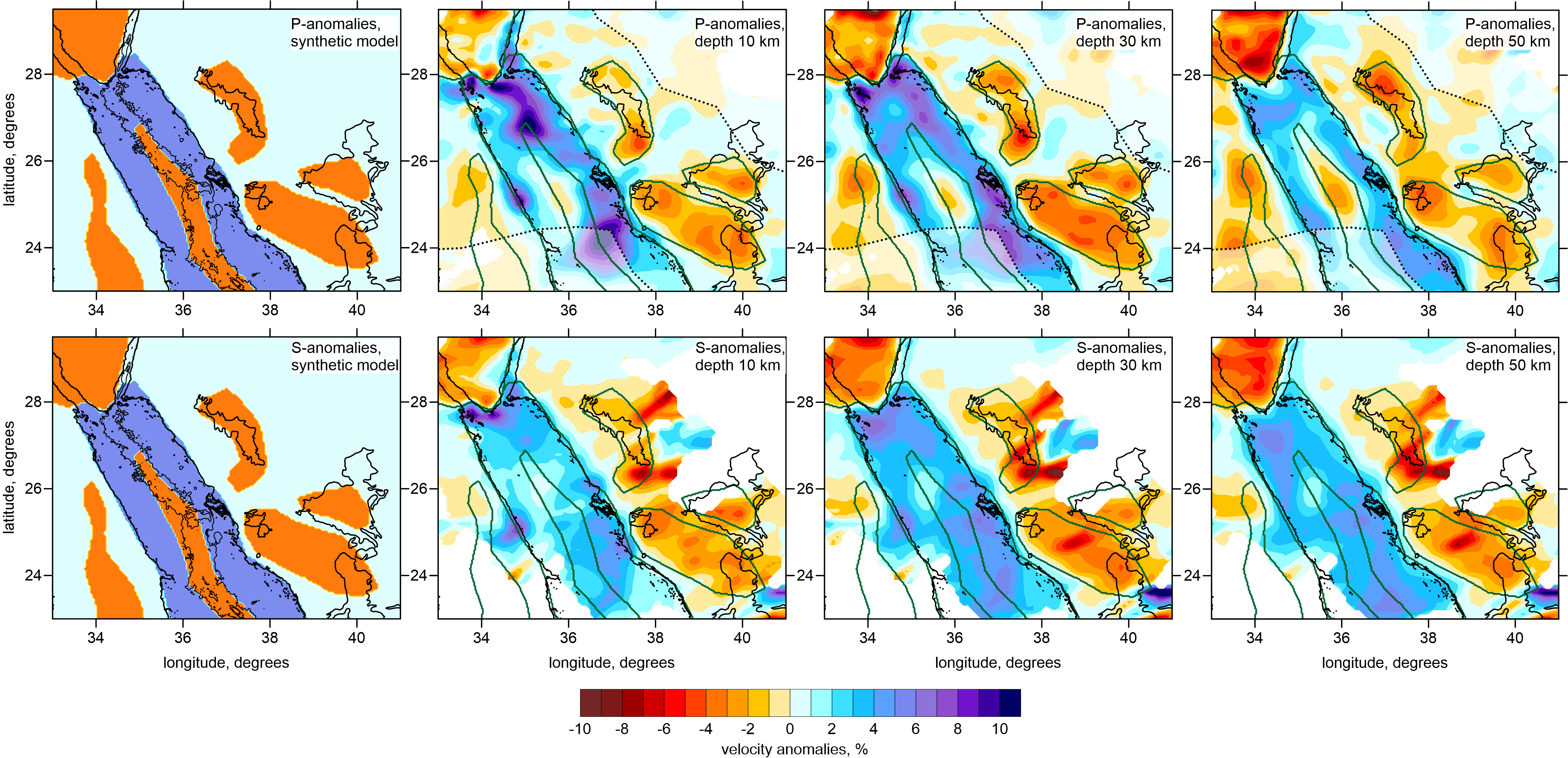


Figure S5. Synthetic test with realistic configuration of the model. The initial model is presented in the left column, as well as in all recovery images with green contour lines. The recovery results for both P and S velocity anomalies are shown at the depths of 10, 30 and 50 km. Note that the anomalies derived in this test are very similar to the results of experimental data inversion. Dotted lines on the P-wave model indicate the resolved area. The images have been produced using the Surfer Golden Software 13 (https://www.goldensoftware.com/products/surfer)


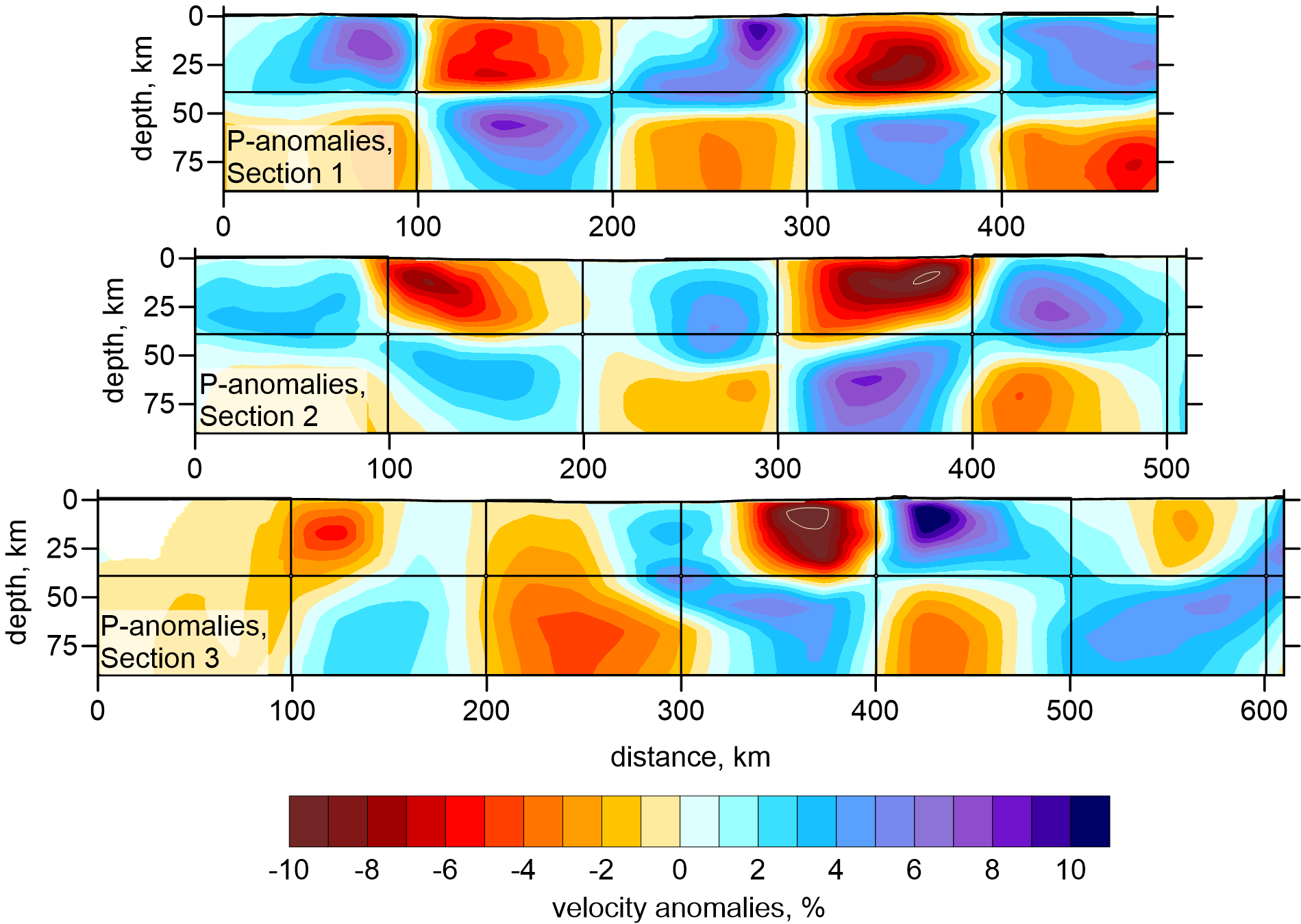


Figure S6. Synthetic test for checking the vertical resolution. The checkerboard models are defined along three profiles, same as used for presenting the main results in Figure 2 of the main paper. Contour lines highlight the shape of the synthetic anomalies. The images have been produced using the Surfer Golden Software 13 (https://www.goldensoftware.com/products/surfer)


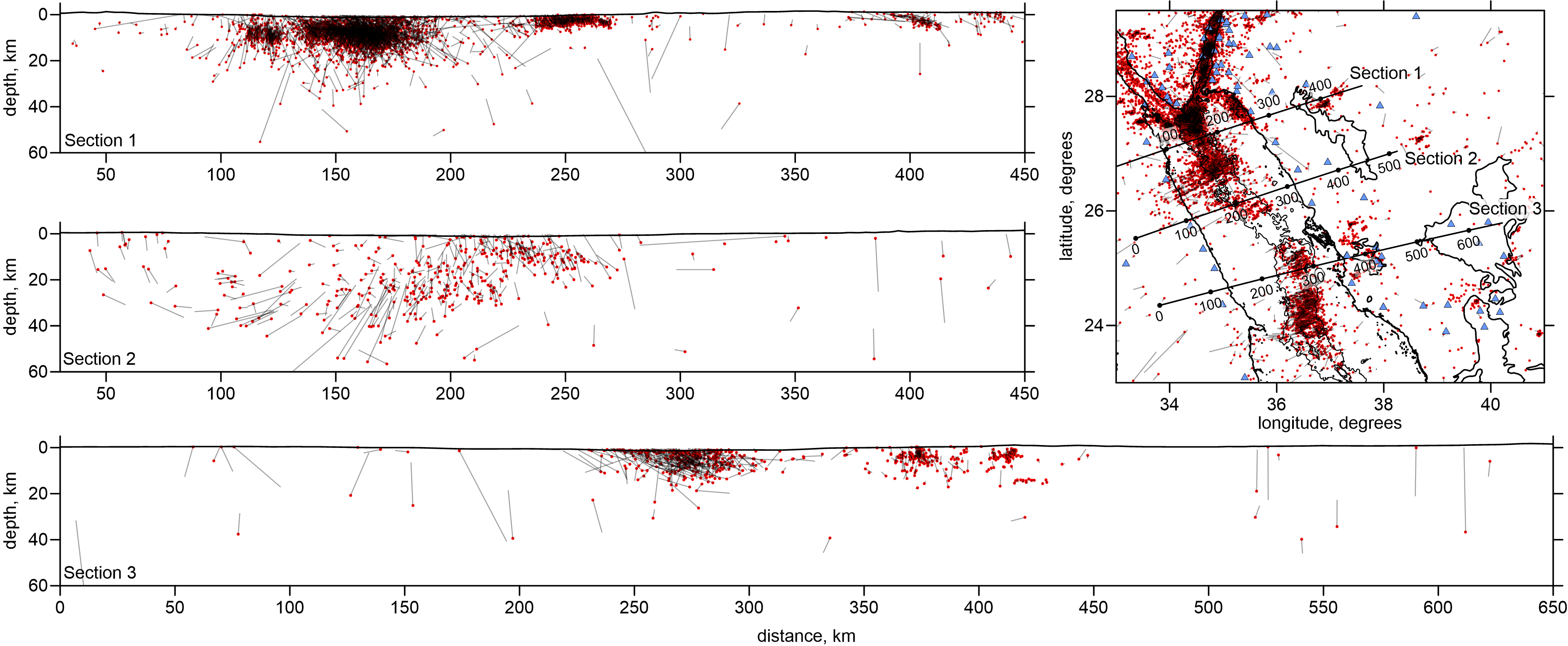


Figure S7. Source mislocations during performing the the checkerboard test (Figure 2) presented in three vertical sections and map view. Red dots correspond to the source locations after five iterations, and the bars indicate to the true source locations. Blue triangles in the map indicate seismic stations whose data are used in this study. The images have been produced using the Surfer Golden Software 13 (https://www.goldensoftware.com/products/surfer)


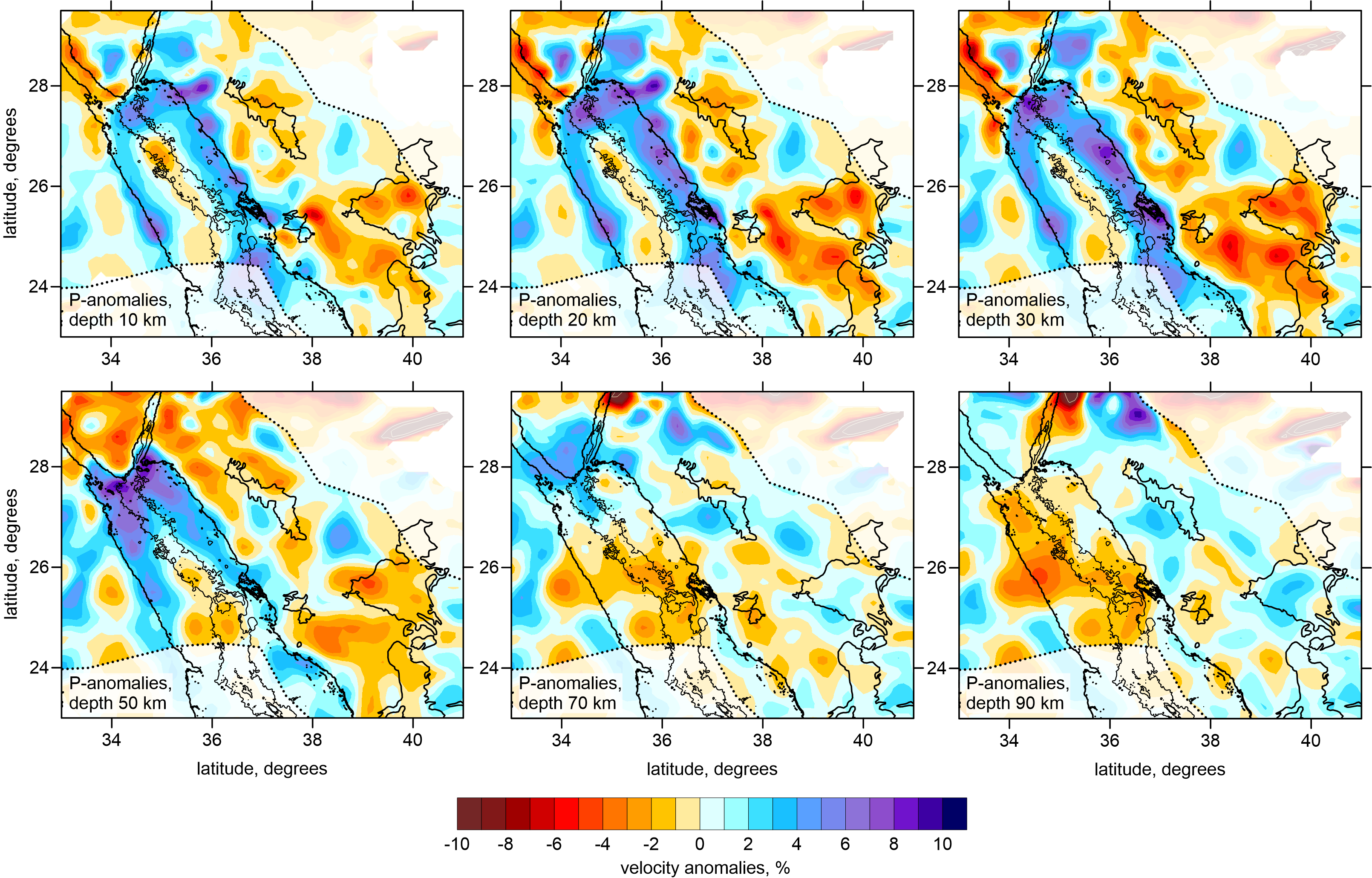


Figure S8. P-wave velocity anomalies obtained from tomography inversion of experimental data presented in six horizontal sections. The contours of harrats are shown in the Saudi Arabian side. Within the Red Sea, the bathymetry is shown with the contour lines starting from 1000 m and with the interval of 500 m. Dotted lines indicate the resolved area. The images have been produced using the Surfer Golden Software 13 (https://www.goldensoftware.com/products/surfer)


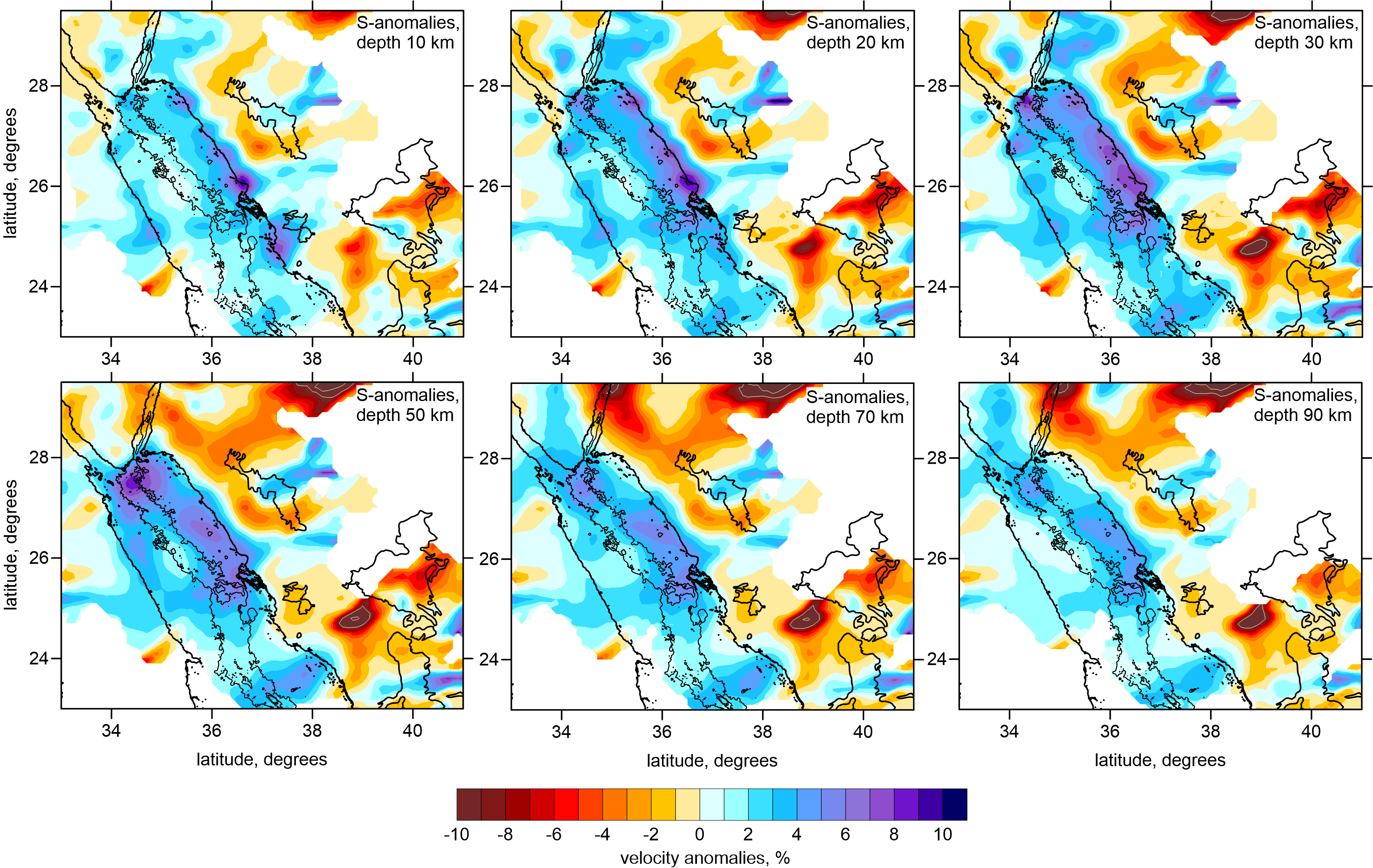


Figure S9. S-wave velocity anomalies obtained from tomography inversion of experimental data presented in six horizontal sections. The contours of harrats are shown in the Saudi Arabian side. Within the Red Sea, the bathymetry is shown with the contour lines starting from 1000 m and with the interval of 500 m. The images have been produced using the Surfer Golden Software 13 (https://www.goldensoftware.com/products/surfer)


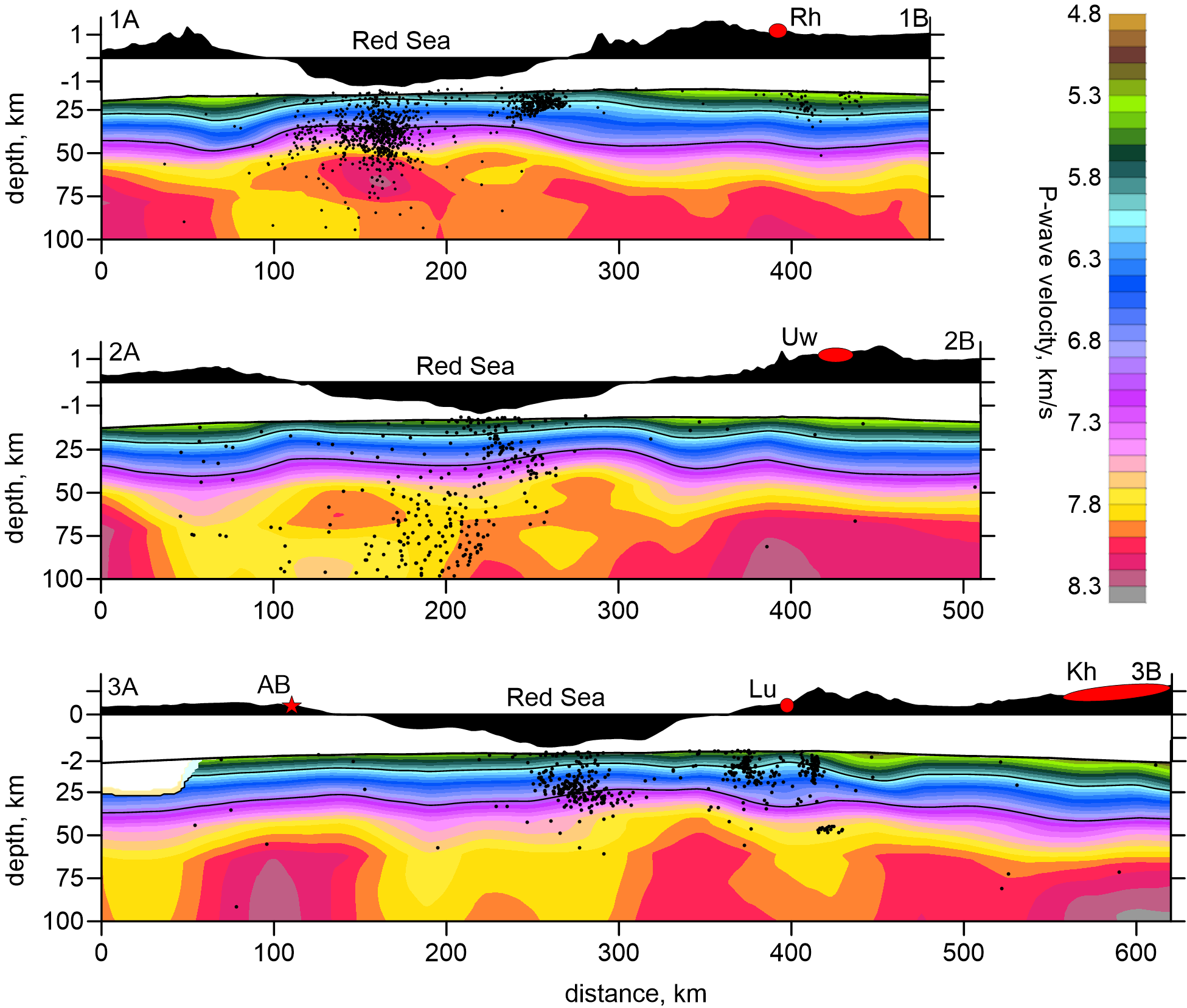


Figure S10. P-wave velocity in three vertical sections, same as shown in the main paper in Figure 2. Above each of the vertical sections, the topography/bathymetry along the profile is shown. The locations of the main harrats are shown in red ellipses as follows: Rh – ar Rahah, Uw – Uwayrid, Lu – Lunayyir, Kh – Khaybar, and Rt – Rahat. Black dots indicate the locations of the events at distances less than 40 km from the profiles. The images have been produced using the Surfer Golden Software 13 (https://www.goldensoftware.com/products/surfer)
